# Taking the EEG Back Into the Brain: The Power of Multiple Discrete Sources

**DOI:** 10.3389/fneur.2019.00855

**Published:** 2019-08-20

**Authors:** Michael Scherg, Patrick Berg, Nobukazu Nakasato, Sándor Beniczky

**Affiliations:** ^1^Research Department, BESA GmbH, Gräfelfing, Germany; ^2^Department of Epileptology, Tohoku University, Sendai, Japan; ^3^Department of Clinical Neurophysiology, Danish Epilepsy Centre, Aarhus University Hospital, Aarhus, Denmark

**Keywords:** EEG, epilepsy, evoked potentials, source space, source montages, dipole source localization, multiple discrete sources, linear transformation

## Abstract

**Background:** In contrast to many neuroimaging modalities, clinical interpretation of EEG does not take advantage of post-processing and digital signal analysis. In most centers, EEG is still interpreted at sensor level, exactly as half a century ago. A major task in clinical EEG interpretation is the identification of interictal epileptiform discharges (IEDs). However, due to the overlap of background activity, IEDs can be hard to detect in the scalp EEG. Since traditional montages, like bipolar and average reference, are linear transformations of the recorded channels, the question is whether we can provide linear transformations of the digital EEG to take it back into the brain, at least on a macroscopic level. The goal is to improve visibility of epileptiform activities and to separate out most of the overlap.

**Methods:** Multiple discrete sources provide a stable linear inverse to transform the EEG into source space with little cross-talk between source regions. The model can be based on a few dipoles or regional sources, adapted to the individual EEG and MRI data, or on selected standard sources evenly distributed throughout the brain, e.g. below the 25 EEG standard electrodes.

**Results:** Auditory and somatosensory evoked potentials serve as teaching examples to show how various source spaces can reveal the underlying source components including their loss or alteration due to lesions. Source spaces were able to reveal the propagation of source activities in frontal IEDs and the sequential activation of the major nodes of the underlying epileptic network in myoclonic epilepsy. The power of multiple discrete sources in separating the activities of different brain regions was also evident in the ongoing EEG of cases with frontal cortical dysplasia and bitemporal lobe epilepsy. The new source space 25 made IEDs more clearly visible over the EEG background signals. The more focal nature of source vs. scalp space was quantitatively confirmed using a new measurement of focality.

**Conclusion:** Multiple discrete sources have the power to transform the EEG back into the brain by defining new EEG traces in source space. Using standard source space 25, these can provide for improved clinical interpretation of EEG.

## Introduction

**Figure 1:**
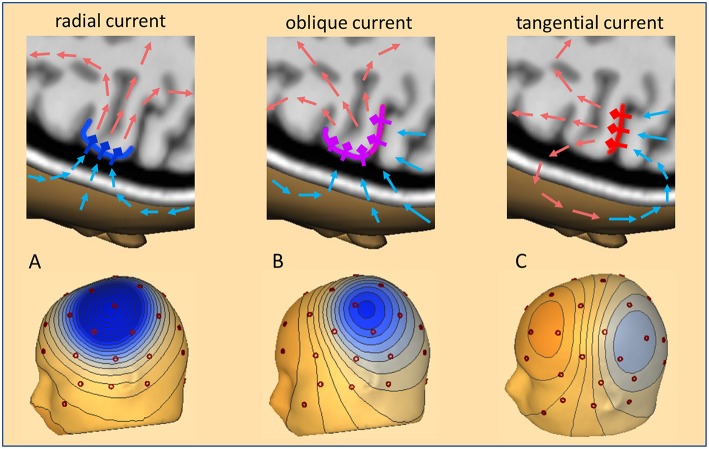
Cortical currents, volume currents, and scalp topography. Three cases of IED current inflow into a focal cortical patch are illustrated: **(A)** radial (dark blue), **(B)** oblique (pink), **(C)** tangential (red). Pyramidal cells and their apical dendrites are symbolized by diamonds and thick outward bars. A subset of return current loops is depicted by arrows in light red to illustrate where they create positive and light blue where they create negative voltages. Depending on the net orientation of the cortical patch, the zone of maximal inflow from scalp into depth shifts from above the patch **(A)**, to more posterior **(B)** and fully posterior **(C)**. These currents create the typical 3D-voltage topographies on the scalp related to a focal IED at the cortical convexity **(A)**, in the depth of a sulcus **(C)**, and, the more common case of an oblique net current involving both superficial and sulcal cortex **(B)**.

The dipolar activities of the different brain regions appear widespread over the scalp and generate a complex overlap in the EEG. Although the radially oriented activities of the cortical convexity are more prominent in the EEG, two thirds of the cortex lie in fissures and lead to widely distributed topographies on the scalp as illustrated in Figure 1. When trying to localize a focal interictal epileptiform discharge (IED), we are looking for a negative peak in the scalp EEG based on the fact that the pyramidal cells at the crown of a gyrus are depolarized at their apical dendrites. Thus, the primary, intracellular currents flow radially into the depth parallel to the cortical columns. The associated return currents form closed loops (Figure 1). Only a small portion of the current passes the skull and returns along the scalp. This creates the positive peaks on the scalp at the maximum exit zone (illustrated by the light red arrows in Figure 1) and the negative peaks at the maximum reentry zone (light blue arrows).

With an ideal radial current, the scalp negativity is exactly over the superficial cortical generator (Figure 1, left). If the depolarization occurs on one side of a sulcus that goes straight into the depth, the associated tangential current creates a scalp topography with symmetric negative and positive poles perpendicular to the sulcus, with the positivity on the active side (Figure 1, right). Normally, focal IEDs involve both sulcal and superficial cortex or deeper parts of sulci. The resulting net orientation of the primary current is oblique. The associated negative and positive peaks are unequal and somewhat shifted away from the active cortex (Figure 1, middle). In fact, the negative peaks can be far away from the active cortex especially if the focus is deeper in the brain, leading, for example, to the so-called “paradoxical” or “false” lateralization over the wrong hemisphere. Thus, a key problem of interpreting the EEG is its crucial dependence on the orientation of the active cortex.

**Figure 2:**
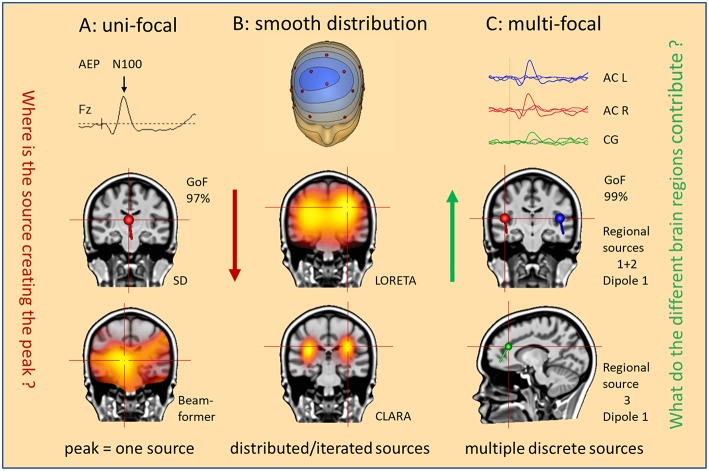
What is our hypothesis? Three hypotheses are illustrated as mental starting points to understand the origin of the N100 peak of the LAEP. **(A)** Uni-focal: a single dipole (SD) or beamformer will localize to an equivalent center in the middle of the brain. **(B)** Smooth distribution: activity appears widely distributed in the Brain (LORETA); separate foci can be better isolated by iteration of the smeared images (CLARA). **(C)** Multifocal: what do the different brain regions contribute? Multiple discrete sources (RS1–RS3) model the three regions (AC L, AC R, CG) involved in the generation of this LAEP. The answer is the source waveforms shown on top.

The other problem is the traditional concept of the EEG. When observing a spike in the EEG, our primary thought is: Where is the source of this spike? Intuitively, we assume a single source for a prominent spike or a peak in an evoked potential (Figure 2). However, considering the complex overlap on the scalp, we might pose a different question: What do the different brain regions contribute to cause this spike or peak? This new approach implies that we can confirm the existence of a focal origin only, if we can show that no region in the brain except one is contributing substantially. This is the basic concept of reverse source imaging (RSI) using multiple discrete sources (MDS), as detailed below.

Obviously, we cannot uniquely reconstruct the activity of all pyramidal cells from the few recorded EEG channels, not even of all gyri and sulci in the brain. This is the so-called inverse problem of EEG, i.e. to estimate the regions in the 3D-brain that contribute to the 2D-voltage distribution on the scalp surface. Figure 2 illustrates different approaches how to solve the inverse problem. Take, for example, the prominent N100 peak of late auditory evoked potentials (LAEP) at mid-frontal electrodes. Using a single dipole, you localize into the middle of the brain with high goodness of fit (GoF: 97%), but not bilaterally into the auditory cortices (AC) as expected (1, 2). The assumption of a single source is not valid. Similarly, beamformers mis-localize to one equivalent center (Figure 2A) unless you assume two symmetric beams pointing into each hemisphere. Distributed source models, on the other hand, use thousands of equivalent dipoles in the brain volume, or hundreds in the cortical gray matter. This requires additional mathematical, non-physiological assumptions. For example, smoothness in source space is used in LORETA (3). CLARA, i.e. LORETA applied recursively (4, 5), separates two foci around the right and left auditory cortices in this LAEP elicited by auditory stimuli of varying intensity (6). However, foci in distributed source images are smeared and shift over time and the small activity of cingulate gyrus (CG), imaged by the MDS model in Figure 2C, was not detected.

MDS models are an alternative to project the scalp data into the brain, here onto three fixed regional sources bilaterally in AC and in CG. Prior to discussing this approach in detail, we need to lay out viable concepts of source space and linear transformations to take the EEG back into the brain on a macroscopic level. At the same time, we must ascertain that our assumptions are appropriate for the data to be analyzed.

In contrast to MRI or CT, EEG is still interpreted at sensor level. Most clinicians reading EEG inspect only raw data. Although accessing undistorted raw signals is important, reluctance to include signal processing into clinical practice precludes any progress in this field. In fact, clinical EEG has proven to be one of the most conservative fields in medicine, where trainees are still taught exactly the same routines as their tutors were several decades ago.

Therefore, this paper documents how to create a new perspective onto the EEG by taking it back into brain source space. This is achieved by simple linear transformations of the scalp EEG in addition to digital filtering in the time domain. First, we need to outline the concepts of equivalent sources, of local and global source spaces and of linear transformations. Then, we can illustrate how brain source space provides additional insight into IEDs and evoked potentials.

## Basic Concepts of Equivalent Sources and Brain Source Space

### The Local Source Space

**Figure 3:**
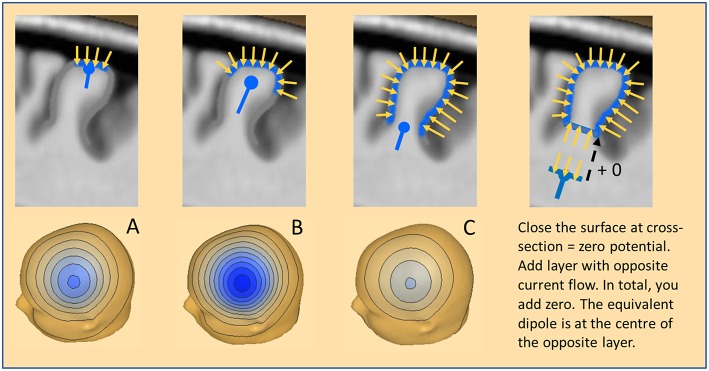
The equivalent dipole. Three cases of cortical patches are illustrated with a resulting net inward current of radial orientation: **(A)** Small patch on the crown of a gyrus. **(B)** More extended patch spreading into the sulci. **(C)** Large patch involving both crown and deep sulci. The equivalent radial dipole sums the local currents along the cortical columns and is moving progressively deeper. For more details see text.

In evoked potentials, the situation appears relatively simple. In the ascending pathways, the local source space is defined by a specific fiber tract leaving a nucleus (7) or crossing a boundary of the volume conductor (8). In sensory cortex, perception occurs in small, circumscribed cytoarchitectonic areas. Thus, the dipole currents of the cortical columns sum up to an equivalent dipole with high precision (Figure 3A). Accordingly, the scalp potential is relatively small (<5 μV). In the case of an IED, the activated area is often much larger, especially in mesial temporal lobe epilepsy with up to 10–20 cm^2^ (9). If we assume a circular shape of the activated cortical patch, this would correspond to a diameter of ~3.6–5 cm and amplitudes >100 μV on the scalp. In extra-temporal-lobe epilepsy, IED amplitudes are often smaller (~30 μV), but this still requires a patch diameter of ~2.5–3.0 cm. Thus, an IED may involve most of the crown of a gyrus (Figure 3B), the whole gyrus (Figure 3C) or even several gyri as well as large parts of sulci.

How do we define an adequate model for this seemingly extended local source space? An equivalent dipole summarizes the dipolar potentials of a small cortical patch (Ø <1 cm) with extreme accuracy. It is located near the center of the patch and oriented parallel to the net current flow of the patch (Figure 3A). If the patch is curved and more extended, the equivalent dipole moves deeper into the white matter (Figure 3B). If an IED extends uniformly around a gyrus (Figure 3C) the equivalent dipole locates at the bottom of the gyrus, deep in the white matter. In all cases, the equivalent dipole lies at the center of an imaginary near-to-planar cross-section of the activated patch. As shown on the right, we can close the activated patch by a cross-section having a dipole density equal to the patch and obtain zero-potential outside, because the net dipolar currents of a closed surface cancel. By mentally adding the same cross-section with the opposite dipole current density, we add nothing and create zero potential from the upper closed surface. What remains is the opposite dipole field of the added near-to-planar cross-section. Thus, the shape of the gyrus above the cross-section has no influence whatsoever on the EEG scalp potential. We only observe the dipolar potentials coming from the equivalent, uniformly active cross-section at the bottom.

What is the magnitude of the negative scalp peak in these three cases having the same radial orientation of the net current flow? The superficial patch A produces a small and focal negative peak; the wider patch B involves a much larger surface leading to higher magnitude. Interestingly, the widespread activation around the gyrus in case C leads to the smallest peak for two reasons: (1) the effective cross-section is small, (2) the equivalent dipole is considerably deeper in the brain. However, the shape of the voltage map in these 3 cases changes only slightly with the increasing depth of the equivalent radial dipole.

What about the accuracy of the forward estimation of the scalp potentials, if we use an equivalent dipole at the center of the patch as our source model instead of the whole dipole sheet at the cross-section? Repeating previous simulations (10) we used 40 standard electrodes and 6 hexagonally arranged equivalent dipoles plus a center dipole below Cz to mimic the scalp potentials of a superficial circular cortical sheet placed at the outer cortical convexity (eccentricity: 80%). The difference in residual variance (RV) between the potentials created by the sheets and a single center dipole was <0.01% for the small hexagons around each dipole, 0.02% for a patch of 5.2 cm^2^ spanned by the 7 dipoles (Ø ~ 2.6 cm) and only 0.17% for a patch of 20 cm^2^ (Ø ~ 5 cm). As expected from the curved surface (Figure 3) the fitted equivalent dipole stayed below Cz and moved into the depth to the level of the cross-section, i.e. to 76% eccentricity with the patch of 5.2 cm^2^ and to 67% with 20 cm^2^. Even in this last, worst case, the observed inaccuracy was more than one dimension smaller than the typical errors of 2–5% when fitting a dipole to an averaged IED.

Thus, in view of the substantial background noise still remaining after averaging, there is no way to estimate the extent of an IED source from the scalp EEG, because source extent is counteracted by the shielding of the brain activity due to the insulating skull and the highly conducting layers of CSF and skin. Conductivities and thicknesses of theses tissues vary greatly between individuals and cannot be precisely rendered from MRI. Less conducting skulls, e.g. in older subjects, and thicker tissues lead to increased shielding, more widespread topographies and deeper equivalent dipoles. If we force distributed dipoles into the cortical folds based on the individual MRI, the extent of activation along the cortical surface is mostly determined by the assumed tissue parameters. Even the amplitude of the scalp peaks is only a crude indicator of the extent, as can be seen from Figure 3 and by comparing large IED amplitudes in children having focal cortical dysplasia with small IEDs in the elderly having large polar areas involved in temporal lobe epilepsy.

**Figure 4:**
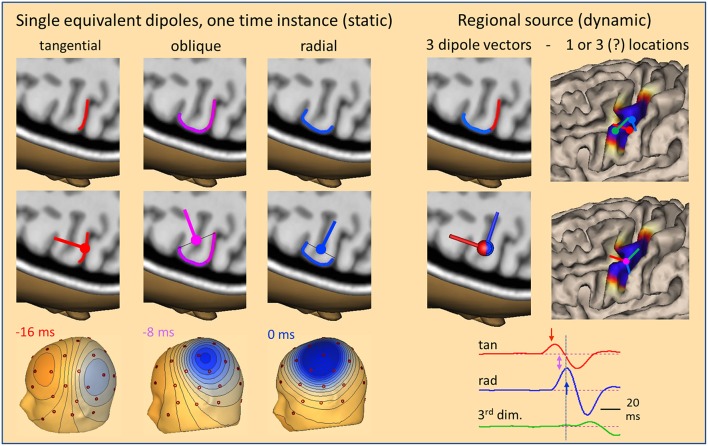
The local source space. Using snapshots at one time instance as in Figure 1, we can define the local source space by a single equivalent dipole (**left**) moving from the sulcus (−16 ms, tangential) into the white matter (−8 ms, oblique) and closer to the surface (0 ms, radial). The changes in location are very small, but orientation changes completely, rotating from tangential through oblique to radial within 16 ms. The cross-sections of the active patch at each time are illustrated by lines. The equivalent dipole is located at their midpoint. An alternative local source space is defined by a regional source (**right**) having 3 orthogonal dipoles at a common center location to describe the 3-dimensional volume currents with high precision and rotational invariance. When rotated appropriately, the radial dipole depicts the superficial and the 1st tangential dipole the sulcal currents. Their source waveforms (right bottom) show the contribution of the local source space to the measured data. For more details see text.

So far, we have only considered radial sources at one point in time. If we look at the evolution of an IED over time, we can take snapshots at different time-points from onset to peak to compare the dipolar scalp maps with the location of the activated cortex and the related equivalent dipole (Figure 4). Given the idealized situation that an IED starts at the anterior wall of a sulcus perpendicular to the convexity, our snapshots show an equivalent tangential dipole at onset (−16 ms, red), followed by an oblique dipole when superficial cortex becomes involved (−8 ms, pink) and a radial dipole (0 ms, blue) when the activity of the superficial cortex peaks while the tangential, sulcal onset activity is crossing zero after its first peak (cf. related arrows along source waveforms in Figure 4, right). The equivalent dipole is always located at the center of the smallest cross-section that is equivalent to the complex patch of activated cortex. Location changes minimally while dipole orientation is changing continuously.

This type of model is called a moving dipole model. However, the brain structures are fixed and not moving. This is taken into account by the so-called regional source model (2, 10, 11). The regional source is fixed to the local brain structure by assuming one equivalent location in the depth of the gyrus. This model is more robust, because it assumes only one location over time and not a new location at each point in time. Allowing for a small error in location, e.g. in source depth, is not critical, because the resulting change in scalp topography is minimal as shown before. Dynamics is modeled by 3 time-varying dipole vectors describing the local current flow over time in any direction (Figure 4, right). Thus, by having 3 dipole vectors at a common location oriented, e.g. along the orthogonal x,y,z-axes of the AC-PC or Talairach coordinate system, dipole current in any direction is fully projected into this local source space, i.e. one calculates the dipole orientation and magnitude at each point in time and projects this onto the 3 axes.

The main advantage of the regional source is that the axes can be rotated to obtain fixed orientations to match the local anatomy (Figure 4, right bottom) without any change in the resulting model of the observed scalp waveforms. Thus, we can choose the first dipole to be tangential in order to estimate the sulcal IED onset activity oriented into and perpendicular to the posterior wall of the active gyrus while the 2nd dipole is oriented radially into the depth to model the superficial cortex-negative IED. The third dipole serves to image the local current along the gyrus—often quite small in evoked potentials and IEDs. Thus, we obtain 3 source waveforms (12) for each regional source in the brain. When oriented appropriately, we can identify the local area of earliest onset and the local propagation, e.g. from sulcus to surface, by inspecting the source waveforms (Figure 4).

Could we try to localize the sulcal, superficial and 3rd dipoles independently (Figure 4, right top)? We would have to find time-points when the signal from one region is large with zero overlap from the others, but this rarely happens. For example, when trying to fit the tangential onset, activity is weak and noisy and localization is unstable. If we try to fit a later activity, overlap from the previously activated surfaces will bias localization. In contrast, if we fit one common location to the time course of the IED, this is much more stable, because the regional source model needs less parameters. Assume an averaged IED with 25 channels and 20 time samples involving just one gyrus. The 3 locations and 3 orientations of a moving dipole can be estimated at every time point to reduce the number of parameters from the measured 500 values to 120. Using a regional source, the estimate is more robust and needs only 63 parameters, i.e. 3 for location and 20 for each source waveform. However, the greatest benefit of the oriented dipole vectors of a fixed regional source is that they provide a straight-forward linear transformation of the scalp signals into the local source space matching functional anatomy as shown below.

### The Global Source Space

**Figure 5:**
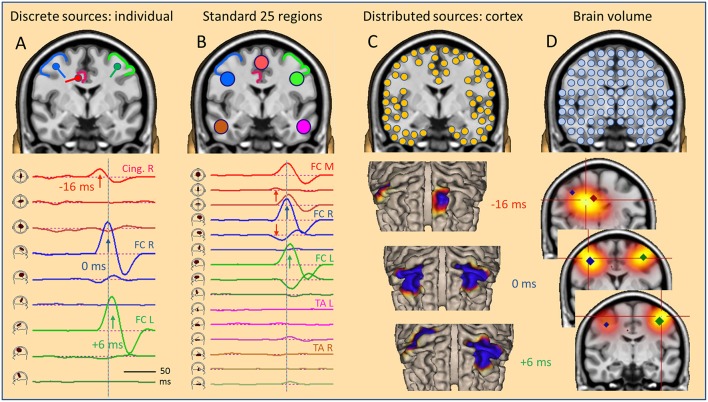
The global source space. EEG/MEG can be modeled by: **(A)** multiple discrete sources (MDS) adapted to the individual data, **(B)** standard source regions, grossly distributed within the brain, **(C)** distributed sources confined to the gray-white matter boundary of the cortex, **(D)** sources distributed throughout the brain volume. Results in a simulated case of an IED propagating from right CG (**A**, red) to right (**A**, blue) and left frontal (**A**, green) superficial cortex depend severely on the choice of global source space. With a small number of sources **(A,B)**, temporal dynamics is revealed by the source waveforms (bottom). However, the large number of sources in distributed models requires the display of a time series of images, either on the cortical surface (**C**, cortical CLARA) or in the brain volume (**D**, CLARA). Typically, distributed source images are calculated independently from one time point to the next. Thus, foci appear smeared and moving over time. Their extent depends more on scaling and other factors used to create the images than the underlying extent of active cortex. For more details see text.

A simulation is presented in Figure 5 to show how to extend the local source space concepts into the global source space of the brain. Assume a focal IED starting out in the right mesial frontal lobe. After averaging, some EEG background noise is still present. The activity is modeled by a single dipole (red) pointing into the right CG almost horizontally and slightly downward. As activation pattern, a biphasic spike has been assumed with the first peak modeling the surface negativity, i.e. the depolarization of the pyramidal cells by the current flowing into a small cortical patch in right CG. Using a regional source with 3 orthogonal dipoles, the first dipole takes up all the early activity peaking at −16 ms after orienting the local source space along the dipole field at this onset peak.

Next, we assume that the local IED is propagating to the right and then to the left lateral frontal cortex (FC). Propagation via the connecting fiber tracts is faster to ipsilateral (right, 16 ms) than contralateral (left, 22 ms) due to the shorter ipsilateral pathway. Again, the local IEDs in these propagated regions show biphasic patterns. These might be spread out a bit more in time due to temporal dispersion and larger in amplitude due to recruiting larger cortical patches in the propagated regions. Again, we place 2 regional sources into the propagated right and left lateral frontal regions and orient each source at its maximum. Now, the first dipole of each source is depicting the biphasic source waveform of the IED in each region. The temporal sequence of deep onset and subsequent propagation to ipsi- and contralateral is fully reconstructed by the source waveforms of this MDS model.

**Figure 6:**
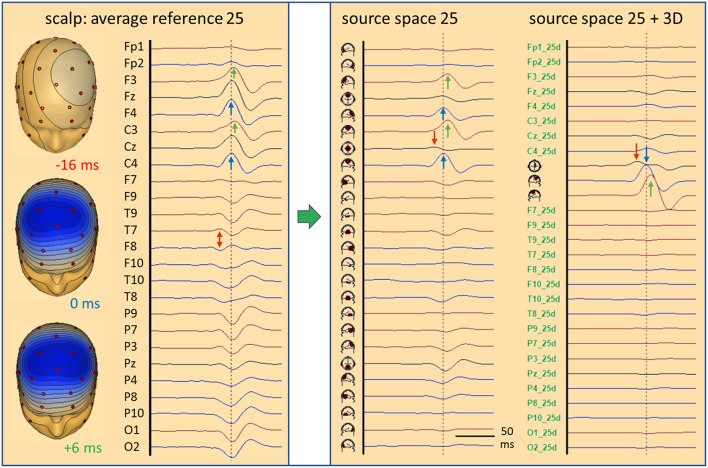
Standard source space 25. In the simulated case of a propagating frontal IED (cf. Figure 5), transformation from scalp (**left**) into source space (**right**) is illustrated. The same sequence of channels from Fp1 to O2 is used to compare the scalp potential with the radial current below each electrode (source space 25). Source channels F9–10 and T9–10 have specific orientations (see text). Time scale is expanded to visualize the different latencies; amplitude scales are relative to enable comparison. The weak onset activity of CG (red arrows, −16 ms) is barely seen on the scalp, but has an oblique dipolar map. Peak latencies appear earlier in the right frontal-central channels F4 and C4 (blue, 0 ms) than left F3 and C3 (green, 6 ms). In source space 25, the activity appears more focused to the frontal-central region with much less cross-talk to other regions. The superficial right and left frontal activities appear better separated (blue, green). On the right, source space 25+3D shows the combination of the 3 simulated source dipoles with the 25 standard regional sources. This isolates the IED and the flat signals of the 25 standard regions document that they are not involved in the averaged IED. The near-to-tangential onset (red arrow) is seen more clearly now as compared to source space 25. For more details see text.

The scalp signals, simulated at 40 standard electrodes (Figure 6), show severe overlap with a broad mid-frontal negative peak shifting from right to the left between 0 and 6 ms. The deeper tangential onset activity (−16 ms) is quite weak. It is barely seen in the scalp waveforms (Figure 6, red arrow) but clearly visible in the 3D onset map. As illustrated above for oblique dipoles in fissures, we observe a “false” lateralization of the negativity over the left hemisphere at a location to where the negative pole of the red dipole is pointing (Figure 5). The overlap at the scalp has been separated by projecting the 40 scalp signals into this individual source space defined by the 3 regional sources, fixed to the anatomy and not moving over time. The first dipole of each regional source has been oriented to the maximum activity of each source while the other two dipoles show no significant activity along their spatial orientation. They only reflect EEG background noise and some small cross-talk due to slight smoothing of the inverse linear operator used to limit the influence of noise (cf. Methods).

What do we see when using 25 fixed regional sources distributed evenly throughout the brain placed, e.g. below the 25 standard electrodes (13) of the International Federation of Clinical Neurophysiology (IFCN) at an eccentricity of 70%? Five of these sources lie in a para-coronal frontal plane: frontocentral (FC) mesial, right, left, temporal anterior (TA) right and left, approximately below Fz, F4, F3, F10, F9 (Figure 5B). Their projected activities show the propagated IEDs in the frontal-central source channels FC R and FC L with correct peak timing. Since the real sources were more superior, FC M sums up a part of both activities to compensate for the inaccurate localization. The onset activity in the depth (eccentricity 38%) is barely seen, but picked up correctly by the tangential, 2nd dipole of FC M. It is reduced in amplitude, because it is shared with other nearby sources, mainly with the 2nd, tangential dipole of FC R. Thus, we could state correctly that the weak onset activity is coming from between and below FC M and FC R. Less cross-talk is observed when looking selectively at the 25 radial source activities below the 25 standard electrodes. This subset of the transformation of the 40 scalp signals into the standard 25 brain regions, i.e. into 75 signals, 3 for each regional source, shows deblurred and more focal signals in the regions near the real sources as compared to the 25 average-referenced signals at the scalp (Figure 6, middle).

When combining the 25 regional sources with the simulated source dipoles into a mixed MDS model, one can construct a specially weighted inverse transformation that renders the 3 source activities correctly and has only a very small cross-talk to the nearby standard sources (Figure 6, right). Again, the deeper onset activity is more attenuated than in the individual MDS model with 3 regional sources (Figure 5, left), since the 25 standard sources act like a partially shielding dipole layer at 70% eccentricity. Yet, this example illustrates one powerful aspect of MDS: The 25 regional sources act as additional probe sources and their small source waveforms document that no significant activity is contributed to the averaged IED by all the other brain regions. How shall we find the active, contributing regions in the brain? This is the critical point of MDS models to be discussed further below.

A different global source space is defined, if we distribute many equivalent sources evenly either throughout the brain or along the cortical surface (Figure 5). In a distributed source model, each brain voxel or cortical patch is modeled by an equivalent dipole. Typically, the current distribution is estimated independently at each point in time and displayed in the brain volume or on the cortical surface. The few scalp potential values−40 in this example—are converted into ~500–5,000 color values of a 3D- or 2D-image (Figures 5C,D). This under-determination requires a mathematical constraint like minimum norm or smoothness is space to obtain an image (14). To reduce the smearing of foci (Figure 2, LORETA), images can be iterated to become more focused. For example, after a few steps using CLARA (4, 5), foci became either separated (Figures 5C,D, 0 and 6 ms) or lumped together into an intermediate focus in the brain volume (Figure 5D, −16 ms). When source space was restricted to the cortical surface, however, foci were incorrectly projected to different gyri along the poles of the underlying deep dipole field (Figure 5C, −16 ms).

Using three different types of global source spaces in this simulated example, the following effects were observed:

- Individual regional sources fixed to the anatomy accurately reconstructed the source activities of the few generating cortical areas.
- Standard source space 25 with regional sources covering the whole brain provided a gross overview over magnitudes and patterns of activity in the brain.
- Distributed sources provided smoothed images on the cortical surface or in the brain volume. Some locations and the moving of foci were incongruent with the simulation.

## Transformation Into Source Space: AEP and SEP as Teaching Examples

The recipe of how to take the EEG back into the brain, i.e. how to calculate the linear inverse, will be detailed in section Materials and Methods. The inverse differs between distributed sources and individual or standard source spaces based on MDS. Whereas distributed models use just one regularization parameter to invert the lead-fields in data space (14), inversion in source space allows for specific regularization of each source to make the inverse stable and exempt specific sources from smoothing (Figure 6) or to remove, e.g. ECG artifacts completely (15, 16).

### Auditory Evoked Potentials (AEP)

**Figure 7:**
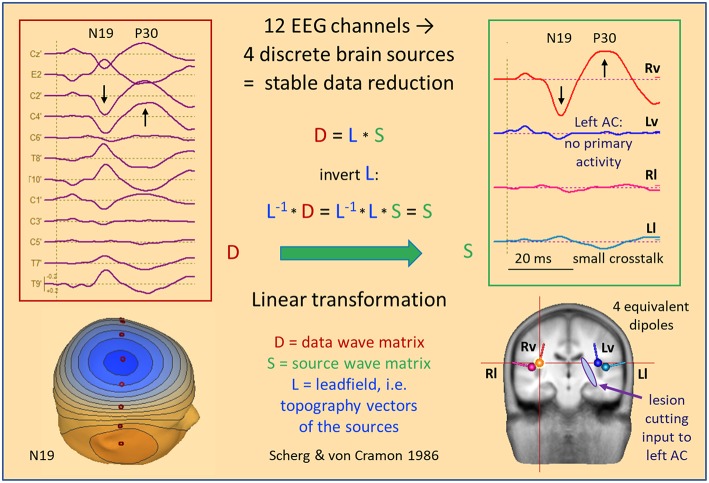
MAEP of a patient with a white matter lesion cutting the input to left AC (2). The distance between scalp traces corresponds to 0.6 μV and transforms into 10 nAm between source waveforms. Filter settings: 10 Hz forward to 200 Hz. The 12 EEG channels in the coronal plane show a right sided dipolar map with the negativity of N19 near the vertex. MAEP scalp potentials are displayed positive up and dipoles with the orientation outward of the cortex—opposite to IEDs—to display P30 upward in the scalp and source waveforms. The linear transformation into standard AEP cortical source space reduces the data to the vertical (Rv, Lv) and lateral (Rl, Ll) activities of the Heschl's gyrus on both sides. Thus, it is immediately apparent that the primary activity of left AC is completely abolished by the lesion whereas the healthy right AC exhibits the typical N19-P30 pattern of the MAEP.

Figure 7 shows data and source waveform matrices to illustrate how and why we can take the EEG back into the brain on a macroscopic level. The middle latency auditory evoked potential (MAEP) of a patient with a deafferented left auditory cortex (AC) appears widespread over the scalp (2, 17). The signals along a coronal chain of 12 electrodes—perpendicular to the supra-temporal plane and lateral surface of the temporal lobe—are linearly transformed to estimate the 4 currents flowing out of AC in vertical and lateral directions (2, 18), i.e. opposite to the inward orientation of IED dipoles. Despite the widespread distribution over the scalp, the source waveforms show that the vertical N19-P30 activity only arises from right AC (Rv). The deafferented left AC (dipole Lv) does not exhibit any primary activity. The lateral, radial activities were small in this case and showed only a weak cross-talk to the left (Ll). Evidently, fixed dipoles associated with specific functional areas in the brain are needed to create such a linear source reconstruction—silent cortex cannot be localized.

The 4 equivalent dipoles were seeded at the mean fitted locations of 42 subjects (19). They were used to calculate the forward solution, i.e. the topography, or leadfield matrix L having 4 columns and 12 rows. Each column contains the voltages calculated at the 12 electrodes in a 4-shell head model using unit currents oriented along one of the 4 dipoles. Using simple linear algebra, the pseudo-inverse of the L-matrix is used as linear operator to transform the scalp data matrix D into the brain source wave matrix S. This macroscopic transformation is stable and unique because the number of signals is condensed from 12 into 4. The 4 inverse vectors have an important property: They render 100% of the source signal they represent, but 0% of the other sources (20). Thus, the first source waveform shows the N19-P30 component of the intact AC, while the second source waveform reveals the loss of activity in the deafferented AC, because there is no cross-talk from dipole source 1 to the location of any of the other 3 sources and vice versa.

The MDS model of the LAEP shown in Figure 2C was created assuming multiple discrete foci by seeding a symmetric pair of regional sources bilaterally into AC (2). After orientation, their first dipoles depicted the vertical P50-N100 complex of the LAEP while their second dipoles showed only a small radial N150. No source components along the Sylvian fissure were seen to rise above the EEG background remaining after averaging. This residual noise was shared by all source waveforms. At high stimulus intensities, a prominent additional component arose around 115 ms and was localized to CG using the grand average LAEP (6). Therefore, a third regional source was seeded at this anterior mid-line location to check the activity of this region at low intensities. There was a peak of activity around 115 ms, smaller than N100, but clearly dissociated in latency and shape from the AC source waveforms after orienting the source to this peak (Figure 2C).

The dipole topographies of this MDS model defined a unique linear transformation of the 32 scalp signals to reconstruct the dynamics of the 9 source activities in the 3 regions. Interestingly, this model explained only 2% more of data variance at the peak of N100 (GoF 99%) as compared to a single dipole in the middle of the brain (Figure 2A), because of the similar scalp topographies of the almost parallel vertical currents in the depth of the left and right Sylvian fissures. However, when taking the temporal evolution into account, the source waveforms revealed the different dynamics of AC and CG during the whole interval from 30 to 150 ms (GoF 98.8%). This separation—possible despite the severe overlap of right and left N100 with N115 at the mid-frontal scalp (Figure 2A)—underlines the power of MDS. The same fixed model with 3 regional sources successfully revealed the different gating effects of P50 in AC and CG despite very poor signal-to-noise ratios (SNR) in the individual data (21).

### Somatosensory Evoked Potentials (SEP)

**Figure 8:**
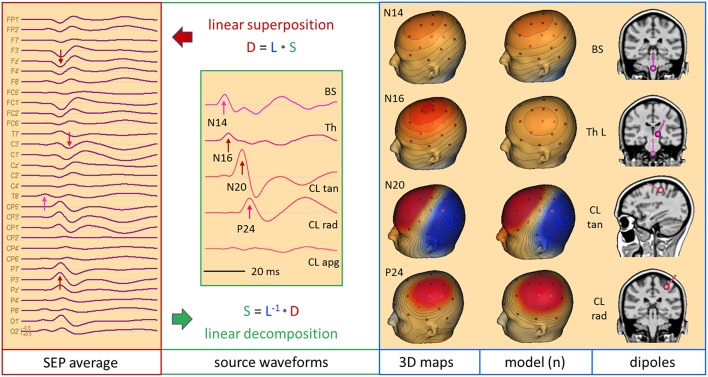
Right median-nerve SEP recorded with 31 channels and high SNR. The distance between scalp traces corresponds to 2 μV and transforms into 30 nAm between source waveforms. Filter settings: 20 Hz forward to 200 Hz. Scalp waveforms show negative up and source waveforms the current flow into the direction of the dipole vector (right). The dipole model consists of a vertical source in the brainstem (BS), an oblique source in left thalamus (Th L) and a regional source (CL) in the contralateral left central hand area (22, 23). The regional source has been rotated to separate the near-to-tangential N20 component (CL tan) from the radial P24 (CL rad). The linear inverse L^−1^ is based on the model topographies of the 5 dipoles shown in column model (n). They are quite similar to the 3D maps at the peaks of N14, N20, and P24. At N16, there is still considerable overlap from N14. L^−1^ reduces the 31 scalp signals to 5 source waveforms showing the separation of the underlying components N14, N16, N20, and P24, labeled by their first peaks. The 3rd dipole of the regional source CL is oriented along the postcentral gyrus (CL apg). Its flat source waveform confirms that (a) no evoked current is flowing along the gyrus, (b) N20 and P24 components fully model the activity of CL, and (c) the model consisting of the preceding 4 dipoles is sufficient to explain and decompose the data.

The median-nerve SEP seems a better candidate for hypothesis A of Figure 2. The N20 peak with its clear tangential dipolar map (Figure 8) invites to localize this peak by a single dipole (24). However, the primary activity of the somatosensory area 3b at the posterior wall of the central sulcus is not where the onset occurs. N20 is preceded by the neural activities of the stimulated afferent pathway. These activities are not over at the latency of N20 and their overlap at the scalp can lead to mis-localization when using only one equivalent dipole. The error depends on the magnitude and orientation of the deep afferent activity that is creating a frontal scalp negativity at the time of N20, thus modifying the dipolar map of N20, and on the distribution of scalp electrodes over the upper and lower head (22, 23).

Figure 8 shows the 31 scalp signals of an SEP average to 10,000 stimuli of the right median-nerve. The 3D scalp maps show the N14 peak of the ascending neural volley and two distinct maps over the contralateral sensorimotor cortex, a tangential map around 20 ms (posterior N20) and a radial positivity around 24 ms (P24). Related deflections are marked in the widespread distribution of the SEP over the upper scalp. As reported (11, 22, 23), the underlying biphasic components associated with N14, N16, N20, and P24 can be separated by seeding a vertical dipole into the brain stem, an oblique dipole in the contralateral thalamus oriented along the ascending thalamocortical tract and fitting a regional source into the contralateral hand area. After rotating the 1st dipole of the RS to the peak of N20 and keeping the 2nd dipole radial outward, we observe the separation of the biphasic N20 and P24 components while no activity is seen along the postcentral-gyrus (3rd dipole). The 32 scalp signals have been projected into SEP source space, i.e. reduced to 5 equivalent source waveforms, by using the inverse linear operator L^−1^ of the 5 dipole topographies.

What can we read from the source waveforms? (A) The 4 peaks reveal the timing of the ascending activity from entering the brain volume (N14) at the foramen magnum (8) over the thalamic output (N16) to the initial cortical activations of area 3b (N20) and superficial areas 1 and 2 (P24). (B) The near-tangential N20 dipole summates the activities of the central sulcus while the radial P24 dipole summates all superficial activities of the contralateral sensorimotor cortex. Further distinctions and separate localizations of P24 and N20 from the 32 scalp channels are impossible. (C) Most importantly, each source waveform shows a flat baseline before the activity starts rising. There is no cross-talk from earlier onto later activities confirming the focal nature of the earlier activities, and the later activities are not influenced by the on-going overlap from the earlier sources. Thus, the SEP can be localized more accurately by having a multi-focal hypothesis (Figure 2C) und using an MDS model in which the deep sources are represented and allowed to be simultaneously active when fitting a regional source into the primary somatosensory cortex, e.g. for presurgical functional mapping (22).

**Figure 9:**
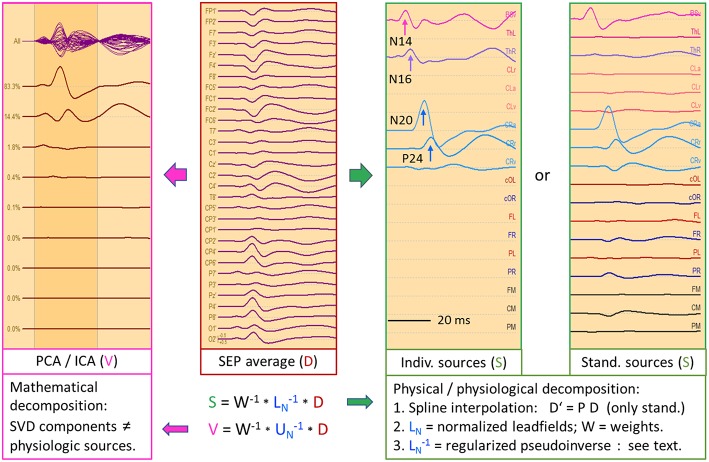
Left median-nerve SEP—same subject, scaling and filtering as in Figure 8. Here, different types of linear decompositions are illustrated. First, one can use the 5 individual sources as in Figure 8, mirror locations and orientation from right to left, and adjust orientations to the peaks of N14, N24, and P24. The separation of components is highly similar to Figure 8, but the generators are now in the right hemisphere, contralateral to stimulation. When using the standard SEP source montage (**right**), one can immediately observe the separation of N14, N20, and P24, although N20 and P24 have not been oriented optimally, so that some activity is shared with the 3rd dipole of CR. The other regions do not contribute to the SEP in this time range and partially shield the deep radial generator of N16 in the right thalamus. This separation in matrix S is enabled by using an appropriate physical and physiological model defined by the leadfields of the sources. If we use a blind, purely mathematical decomposition of the data matrix D, e.g. singular value decomposition (SVD) or ICA, the resulting waveforms V do not show an interpretable decomposition (**left**).

Figure 9 depicts the left median-nerve SEP of the same subject to illustrate the effects of projecting the scalp data into three different source spaces. First, we use the same discrete model with 5 individual sources. The N14 dipole is seeded into the brainstem and oriented to the scalp data while the N16 dipole is mirrored into the opposite thalamus. The added regional source fits into the hand area of the opposite hemisphere and separates the tangential N20 and radial P24 components without showing activity along the gyrus (Figure 9, individual sources). The five source waveforms show a sequence and patterns similar to the right median-nerve SEP.

What happens if we project the scalp SEP into a standard source space constructed to reveal the different source components of the SEP? The source montage in Figure 9 (right) consists of 3 deep dipoles (brainstem and thalamus L & R) with fixed locations and orientations and two regional sources seeded bilaterally into the hand areas in a standard brain with the 1st dipole oriented perpendicular to the central sulcus, the 2nd radially and the 3rd along the postcentral gyrus. Additional sources are placed into secondary somatosensory cortex at the central operculum L & R, bilaterally into frontal and parietal cortex, and into three midsagittal areas to account for overlap from these regions. The N14, N20, and P24 components are very similar to the individual MDS while the deep N16 is quite small in trace ThR due to shielding by the more superficial sources as explained above. The cross-talk to other regions is small and they do not exhibit own activities due to the high number of averages. Again, the other sources act as probes. They confirm the origin of the SEP in brainstem and right sensorimotor cortex and show that the secondary somatosensory areas in both central opercular areas (cOL, cOR) are not activated during fast repetitive stimulation.

This separation is possible, because the different equivalent dipolar sources of the SEP project to the scalp according to the laws of physics. Even when using a simple multi-shell head model, we can predict their model maps on the scalp and reconstruct their source activities quite accurately (Figure 8) provided the maps are not linearly dependent (i.e. one map is highly similar to any combination of the others). In contrast, a purely mathematical decomposition of the data, e.g. independent (ICA) or principal components analysis (PCA), is unable to provide this separation of the underlying physiological components (Figure 9, left).

## EEG: Standard Source Space 25

**Figure 10:**
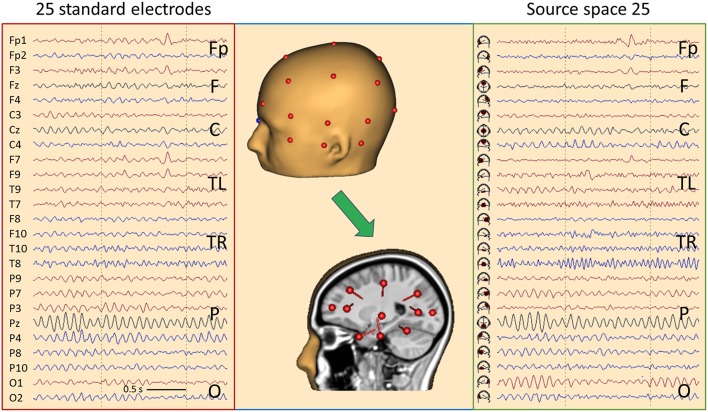
Comparison of the scalp EEG at 25 standard electrodes in average reference (**left**) with standard source space 25 (**right**) using the same sequence of channels in a case with left frontal cortical dysplasia (25). Filter settings optimized for IED review: 2–35 Hz, zero-phase characteristic (26). A spike is seen in second 2, appearing more focal in source space. In addition, the spread of central μ-rhythm onto frontal channels is greatly reduced in source space, making the frontal spike stand out more clearly. In the center the underlying principle of “taking the EEG back into the brain” is illustrated showing the electrodes on the one hand and the underlying source dipoles on the other. For more details see text.

As shown in Figure 6, we can construct a source space using a limited number of sources, for example one source below each of the standard 25 EEG electrodes of the IFCN (13) to get a gross overview of the EEG activities arising from different brain regions. Each of the 25 brain regions below the electrodes is represented by a regional source oriented such that the first dipole points into the adjacent cortex (Figure 10, middle). For most sources, the primary orientation is radial to model the activity of the superficial cortex. Two inferior sources are oriented differently to depict the activities of the temporal pole (F9/F10) and temporal base (T9/T10) in their first dipole source waveforms. Only the first dipoles of each regional source are displayed in source-space montage 25 to obtain a legible overview (Figure 10, right).

For better comparison of scalp and brain signals, electrode and source traces are arranged in the same sequence. Considering the typical sequence of longitudinal and transverse montages (13), the fronto-polar (Fp) and superior frontal channels (F) are displayed at the top, followed by the central channels (C), each group going transversally from left to right. In the middle, the temporal left (TL) and right (TR) groups are displayed going from anterior over inferior to posterior. The temporal groups start with channels F7 and F8, respectively, for convenient perception of temporal polar IEDs. At the bottom, the parietal (P) channels are displayed followed by occipital (O).

Typical EEG rhythms and IEDs are more focused in source space 25, and the source channels where IEDs appear maximally are considerably less contaminated by overlap from other brain regions as compared to the scalp maximum. Figure 10 shows a spike in a case having left frontal cortical dysplasia (25). Source activity is maximal below Fp1 and weaker below F3 and F7. On the scalp, the spike peak appears more widespread from Fp1 to F9. In the preceding 500 ms, the fronto-polar and frontal sources show much less cross-talk of rhythmic activity from the other brain regions. Also, the central μ- and parietal-occipital α-rhythms appear clearer and more focused in source space.

**Figure 11:**
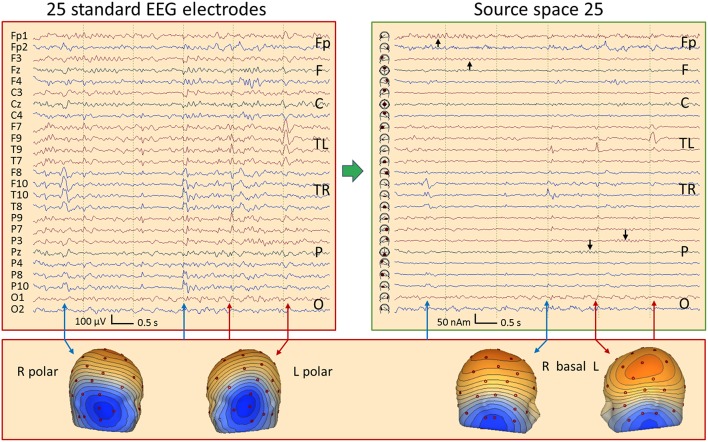
Comparison of the scalp EEG at 25 standard electrodes in average reference (left) with standard source space 25 (right) in a case with bitemporal spiking. Filter settings optimized for IED review: 2–35 Hz, zero-phase characteristic (26). Right (blue arrows) and left (red) polar spikes are seen at the beginning and end of the displayed 6-s EEG segment, most prominent in the inferior channels F10 and F9. In the middle of the segment, IEDs with more complex distributions are seen on the scalp. Transformation into source space 25 provides a much clearer picture: The polar temporal lobe spikes can be identified immediately in the source waveforms below F10 and F9. Basal temporal spikes are seen on the right below T10 (blue) and on the left (red) below T9 (2 spikes emerging clearly, the 1st immediately after the right basal spike below T10). In source space, the temporal spikes appear considerably more focal and emerge much more clearly from the EEG background. In addition, overlap from frontal and parietal EEG rhythms (black arrows) is strongly reduced.

Figure 11 shows 6 s of EEG in a 67-year old female having mesial temporal lobe (mTL) sclerosis with frequent bilateral independent spiking. In source space, four different spike types can be seen almost perfectly focused to the temporal basal and polar traces. One can distinguish immediately whether a spike has propagated from its mesial origin (not visible on the scalp) to the temporal basal or polar cortex. The polar spikes predominate at the inferior-temporal scalp while the basal spikes produce more widespread and smaller scalp peaks. However, scalp voltage maps show the typical polar and basal topographies. EEG background appears much more separated in source space, cf. frontal rhythms in seconds 1 and 2 and parietal rhythms in seconds 4 and 5 (black arrows). The preceding baselines are much clearer in the source traces, because cross-talk from other brain regions is less. Thus, IEDs are more easily detected in source space.

This was confirmed by the new focality measure illustrated in Supplementary Figure 1 and defined in section Materials and Methods. Mean focality of the 65 left-temporal IEDs in this mTL case was 79.5% in source and 65.8% in scalp space (*p* < 0.001, V = 2,104, Wilcoxon two-sided signed rank test). Taking the two most prominent averaged IED types detected by BESA Epilepsy in 25 adults (27), i.e. comparing 50 averaged IEDs of different temporal and extratemporal orgins, mean focality was 71.8% in source and 64.6% in scalp space (*p* < 0.001, V = 981).

The transformation from scalp to brain occurs purely in the spatial domain and does not depend on the temporal dynamics of the EEG. The same linear inverse operator is applied to each EEG sampling point. Hence, a source-space montage is simply a spatial filter combining the recorded scalp signals into a new EEG montage. Instead of subtracting the signals of neighboring electrodes, as in bipolar montages, specific weights are given to each scalp signal to enhance activity coming from the region below the selected electrode while suppressing activities from other regions as much as possible (26). As detailed in Materials and Methods, the linear inverse is stabilized by spline interpolation of the 25 scalp signals onto 81 standard electrodes and slight smoothing of the spatial filter in source space to create 75 source waveforms, i.e. 3 for each regional source. Because the full set of 75 EEG traces is hard to review on a single page, separate source montages with subsets of the 75 signals can be selected to observe, for example, tangentially oriented IEDs in sulci, if these are not apparent in the standard subset of 25 traces showing predominantly the radial activity of each region.

## Propagating IEDs in Standard and Individual Source Spaces

### Frontal IED: EEG and MEG

**Figure 12:**
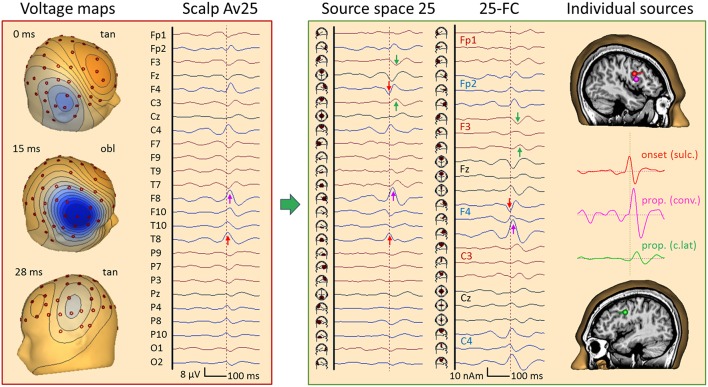
Right frontal propagating IED, 84 averages, EEG results. Filtering: 5 Hz forward to 40 Hz to reveal IED onset (26). In the virtual average-reference montage Av25 (26), the earliest IED peak is seen at T8 (red arrow) followed by F8 (pink). In source space 25, the peak below T8 appears earlier (0 ms) and synchronous with a peak of opposite polarity under F4, indicating an origin between the 2 regions. The corresponding onset map appears tangential, followed by an oblique map with a strong surface negative peak at 15 ms near F8 (pink arrow) and another near-to-tangential map over the left frontal cortex at 26 ms. The left frontal IED appears below F3 and C3 (green) in source space 25. When inspecting all frontal-central channels of source space 25 (25-FC), the strongest peak (15 ms) is seen in the 2nd tangential trace of the regional source below F4 while the earlier onset is seen in the 1st trace. The individual source model needed 3 dipoles to separate the sulcal onset (red, peak at 0 ms) from the larger peak activity (pink, 15 ms) in the same frontal-central region on the right. The 3rd source waveform (green, 26 ms) isolated the IED propagated to the left.

Figure 12 depicts an average of 84 frontal spikes simultaneously recorded with EEG and MEG in a 23 year-old male (16). The EEG shows maximum negativity at F8 reaching out to T8, C4, and F4 with slightly different latencies. An almost synchronous smaller positivity is seen at F3 and F7 while 20 ms later a negativity is barely noticeable at C3 and T7. The maps show a near-to-tangential pattern at onset (0 ms), an oblique pattern at 15 ms with the strongest negativity and a contralateral dipolar pattern at 25 ms.

**Figure 13:**
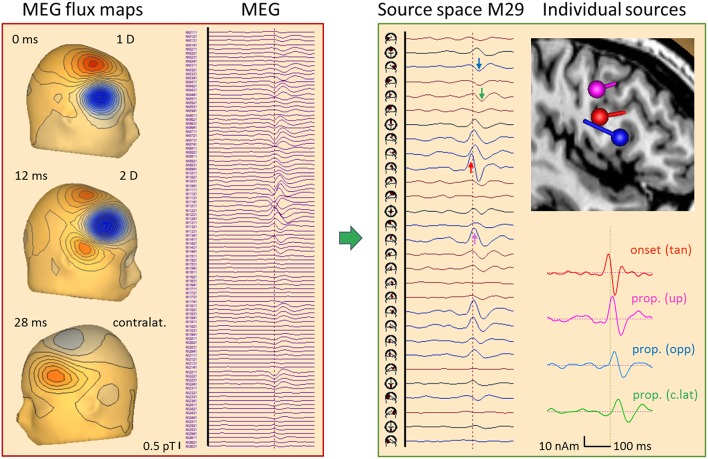
Right frontal propagating IED, 84 averages, MEG results. Time epoch and filtering same as in Figure 12. The 102 magnetometer signals (left) are transformed into source space M29 (16). Each source has 2 tangential dipoles and is rotated to show the maximum tangential activity in each of the 29 regions. The onset shows the largest peak at the right frontal source (red arrow) followed by a delayed peak below C4 (pink) and later peaks below F8 (blue) and F3 (green). A multiple discrete source model with 4 dipoles was sufficient (see text) to separate the onset (red) from activities propagated upwards (pink), forward to the other side of the gyrus (blue) and to contralateral (green), as seen clearly by the increasing delay of the typical biphasic spike pattern in the 4 source waveforms. The flux map at 12 ms illustrates that localization by a single dipole is no longer possible when the propagated activities start overlapping in a complex way.

Source space 25 separated the radial inward activity below F8 (pink arrow) more clearly from the preceding peaks below F4 and T8 (red). Their opposite polarity indicated a tangential activity in between, similar to the contralateral activity occuring later with opposite source peaks below F3 and T7 (green). The fronto-central subset of all 75 source channels (montage 25-FC) showed the strongest regional activity below F4 throughout the onset-to-peak interval (0–15 ms, red and pink arrows). This was followed by the activities of the more central superior (7 ms, below C4), anterior-inferior frontal (15 ms, F8) and contralateral sources (28 ms, F3), as confirmed by MEG (Figure 13).

The individual EEG source space (Figure 12, right) was determined by fitting two regional sources and converting them into separate dipoles oriented to separate onset and peak activities. In the right hemisphere, only the contributing sulcal onset (red) and superficial peak (pink) dipoles were retained in the model together with the contralateral sulcal dipole (green). Their source waveforms provide a dynamic image of the propagation from sulcal to superficial cortex within right frontal cortex and to left frontal with a delay of ~26 ms.

What does the simultaneously recorded MEG tell us about the propagation in this case (Figure 13)? MEG is blind to superficial radial currents, but looks at sulcal activities with higher resolution than EEG. First, we transformed the 102 magnetometers signals into source space M29 while removing ECG artifacts (16). The 29 regional sources were rotated to display the maximum of the two tangential dipole activities in each source trace. Thus, the onset activity of the right frontal cortex appeared very clearly (red arrow), followed by more central (pink) and more anterior (blue) ipsilateral activities. The contralateral left frontal activity (green) peaked ~20 ms later. MEG flux maps showed a dipolar onset pattern (0 ms) followed by a complex, seemingly 2-dipolar pattern at 12 ms and a contralateral dipolar pattern at 32 ms. The individual source space was constructed by fitting 4 dipoles sequentially (11) using the onset phase for dipole 1 and the zero-crossings of the preceding activities for dipoles 2–4 to localize at times with low interference. This revealed an intriguing local propagation pattern in the 4 source waveforms consistent with the individual MRI: From the sulcal onset zone at a rear wall in the inferior frontal gyrus (red dipole, peak at −4 ms: tan), propagation occurred both toward more superior cortex (pink, 10 ms: up) and to the anterior, opposite side of the spiking gyral section (blue, 16 ms: opp). About 20 ms later, propagated activity peaked in contralateral frontal cortex (green, 30 ms: c.lat).

How could such a separation be achieved? The 3 ipsilateral dipoles had different orientations with distinct topographies in the 102-sensor array, and, in this MDS model, their locations were sufficiently different to avoid linear dependence. This would occur when assuming 3 dipoles at exactly the same location, because MEG is sensitive only to 2 tangential dimensions. The source waveforms document that the linear inverse was able to transform the 102 magnetometer signals into 4 source signals with excellent separation of onset and propagated activities for two reasons: (1) There was no cross-talk from one activity to the next. Initially, only the onset region showed activity rising above background; next, the upper source activity started rising while the anterior, opposite wall was still inactive; finally, the 3 ipsilateral dipole activities combined to create the complex superficial flux pattern seen at 12 ms while contralateral source activity just started. The latter was much easier to separate, of course, because its flux map has only little overlap with the maps of the 3 right frontal dipoles. (2) There was no enhancement of background noise in the baselines of the source waveforms prior to the IED. This would appear when coming closer to linear dependence.

The orientations and locations of the tangential onset dipoles in EEG and MEG matched. They point to the same origin at a time when EEG activity was still weak. This underlines that averaging is needed to distinguish IED onset from EEG background in order to localize the triggering onset zone.

### A Case of Myoclonic Epilepsy: Findings in EEG and MEG Source Space

**Figure 14:**
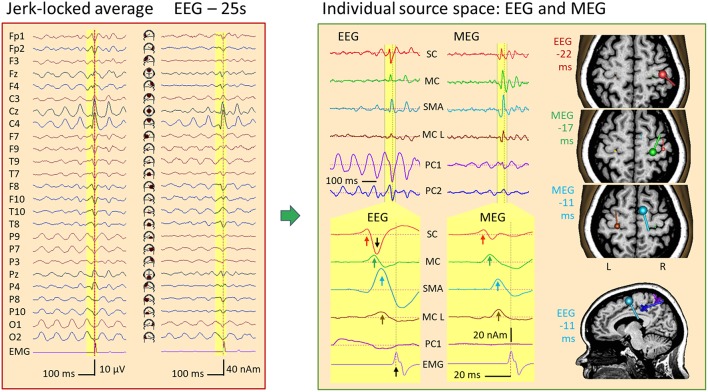
IED in a case of myoclonic epilepsy. Left: jerk-locked average displayed in scalp EEG montage Av25 and source space 25. The motor potential (MP) preceding the left-hand jerk (EMG/FDIL at bottom) is riding on a central 10-Hz EEG rhythm and is most prominent below C4 in source space 25. Right: An MDS model with 4 dipoles and 2 PCA-components modeling the parietal (PC1) and central (PC2) 10-Hz oscillations separates the sharp jerk-locked transient in the wideband EEG and MEG averages from the rhythmic activities (right top). After expanding time scale for better visualization of the yellow pre-jerk epoch, and forward filtering (see text), 4 components could be isolated both in EEG and MEG (for details, see text): IED onset in right somatosensory cortex (SC, red, 22 ms prior to jerk), discharges of right motor knob (MC, green, −17 ms), SMA (blue, −11 ms) and contralateral, left motor knob (MC L, brown, −10 ms). The orientation and strength of source current at these times is illustrated by the dipoles in the slices of the individual MRI, colored accordingly. The posterior mid-line centers of the oscillatory EEG components PC1 and PC2 are shown by crosses in the sagittal slice at −11 ms.

Figure 14 displays the jerk-locked average of 474 myoclonic spikes from a simultaneous EEG-MEG recording of a 36-year-old female with cortical reflex myoclonus. The EMG, recorded from the first dorsal interosseus muscle on the left (FDIL), shows the time of the jerk involving the left-hand digits 1–2. In the unfiltered EEG, widespread rhythmic activities precede the jerk, relatively steady in the occipital channels O1-O2, but progressively building up in central channels Cz and C4 toward the jerk. At C4 and F4, a small spike-like discharge is riding on the rhythmic activity, best recognized in source space 25. It precedes the EMG peak by about 20 ms. Averaging would have completely reduced the rhythmic activity, if it were not time-locked to the jerk. Here, we observed only partial reduction suggesting that the steady posterior 10-Hz rhythm is driving the central rhythmic buildup until the depolarization in the central area is sufficient to gate the initiation of the myoclonic jerk.

Using principal components analysis over the first 3 EEG cycles prior to the jerk, we could define 2 spatial components (11, 15) to model the rhythmic activities with centers of gravity in the midst of parietal (PC1, purple) and central cortex (PC2, dark blue). After using two forward low filters at 10 Hz and 50 Hz to reduce the overlap of the slow activity and expanding the time scale, the averaged IED was localized using 4 dipole sources in MEG (Figure 14 right, bottom): Source 1, peaking 22 ms prior to the jerk of the left hand, was located at and pointed into right somatosensory cortex (SC, red). Source 2 localized at the precentral motor knob (MC, green, −17 ms), source 3 near right SMA (blue, −11 ms) and source 4 near the left, contralateral motor knob (MC L, brown, −10 ms). Finally, dipole orientations were fitted to the data, independently for EEG and MEG, since MEG dipoles only render the tangential and not the full, oblique current vectors as EEG does.

The onset dipole of the IED in SC was tangential with similar source magnitudes of the upward peaks in EEG and MEG source waveforms (red arrows). Similarly, the MC dipole, peaking 5 ms later, was tangential in EEG and MEG with comparable magnitudes. The SMA dipole (blue) showed a predominant radial current 6 ms later. Hence, the SMA peak was considerably larger in the EEG source waveform. The tangential dipole activity of the left motor knob (brown) was relatively weak both in MEG and EEG, but could be localized precisely using MEG. The larger downward peak in the EEG source waveform (black arrow) following the positive onset peak of SC reflects the second phase of the IED with a large inward current into sensorimotor cortex. How much of this predominantly radial activity originated in SC or in MC, could not be fully resolved using EEG due to the closeness of both source regions. MEG, however, rendered only the tangential part of the inflow into the motor knob and showed minimal interference between the SC and MC dipoles because their orientations differed sufficiently in the tangential plane (Figure 14, right).

**Figure 15:**
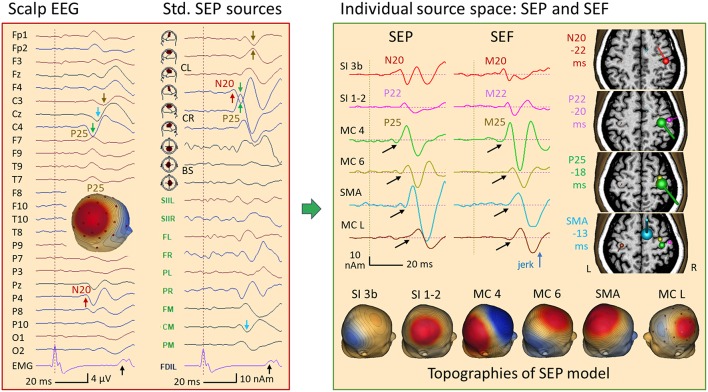
Left median-nerve SEP in a case of myoclonic epilepsy. Left: SEP average in AV25 montage and in SEP source space. Following a weak N20 (P4, red arrow), the giant P25 (28) is seen at C4 (green arrow) and P4. The EMG showed a jerk ~40 ms after stimulation of the left hand and, a weaker jerk of the right hand ~10 ms later. In SEP standard source space, the regional source CR (below C4) displays the N20 in the 1st trace, perpendicular to the central sulcus. Then, it shares the huge P25 component with the radial 2nd source trace of CR (green arrows), resulting in the strong positive peak of P25 between C4 and P4 in the scalp map. A similar giant P25 pattern is seen ~10 ms later in the left central region (CL, brown). Right: an MDS model with 6 dipoles resolved the complex overlap both in EEG and MEG (for details see text): N20 (red) is followed by the more radial post-central P22 (pink), by the pre-central P25 (shared by 2 sources in MC 4, green and MC 6, yellow) and activities in SMA (blue) and the contralateral, left motor cortex (MC L, brown). The peak times relative to the left-hand jerk are given next to the MRI slices used to display MEG dipole orientations and strengths at the peak times of N20, P22, P25, and SMA. Below, the 6 forward EEG topographies of the MDS are depicted. Using their inverse matrix, the 32 scalp EEG channels and, independently, the 122 MEG channels could be taken back into the brain to separate the activities of the nodes of the network underlying this case of myoclonic epilepsy. Black arrows indicate the onset of the later activities that had no obvious cross-talk with the earlier activities.

Figure 15 depicts the SEP average of 375 left-median nerve stimuli in this patient. Here, the EEG rhythms were averaged out due to the asynchrony with the stimulation randomized around 3 per second. In addition to the giant SEP component P25 (28) peaking at C4 and P4 with a latency of 22 ms, the median-nerve stimulus elicited jerks occurring at 40 ms as seen in the EMG (black arrow). The stimulus artifact, seen as the first peak in EMG, was removed from the EEG by 2 spatial components using the SEP source space (Figure 9) as surrogate model to correct the EEG in analogy to ECG-artifact correction (15). This correction defines a linear operator that was applied to the leadfields during source modeling to prevent bias in localization. When inspecting the waveforms in standard SEP source space (Figure 15, left), N20 was seen at a latency of 18 ms in the first tangential trace of the right central sensorimotor cortex (CR). The giant P25 was maximal in the radial trace, but also quite large in the first tangential trace of CR with inverted polarity relative to N20 (green arrows). Thus, P25 showed an oblique dipole pattern with a large positive scalp peak in the 3D-map between C4 and P4. A similar pattern was seen in left sensorimotor cortex (CL) about 9 ms later (brown arrows).

In this patient having myoclonic epilepsy, an enhanced neuronal network was activated following right median-nerve stimulation as suggested by the jerks occuring ~40 ms later in the left hand (black arrow in EMG channel FDIL) and weaker jerks seen in the EMG of the right FDI ~50 ms post-stimulus. MDS analysis of the somatosensory evoked potentials (SEP) and magnetic fields (SEF) revealed six nodes of this network. These involved not only somatosensory, but also right and left motor cortex as suggested by multiple discrete source analysis (Figure 15, right). The first deflection, i.e. N20, localized to the known postcentral area 3b (peaking 18 ms after the stimulus and −22 ms prior to the jerk, red). Adding a more superficial and lateral dipole to model the somatosensory areas 1–2, the P22 activity could be isolated both in EEG and MEG (pink, −20 ms). Two sources were needed to separate P25 from the continuing overlap of the somatosensory activities that had started earlier: a near-to-tangential dipole in the lower parts of the motor knob, likely area 4 (MC 4, green, −18 ms) and a more superficial oblique dipole at the crown of the motor knob (MC 6, yellow, −17 ms). The next two nodes were modeled by dipoles in the SMA (blue, −13 ms) and in the left, contralateral motor knob (−10 ms, brown). This model could be constructed either by seeding the dipoles using the individual MRI and the known anatomical locations of the sensorimotor areas representing the first 2 digits, or by using localizations from MEG and EEG. In both approaches, the key was to optimize orientations such that each dipole source waveform had a flat baseline prior to its onset (black arrows).

## Discussion

Since the 10–20 electrode system was introduced in 1958 (29), reviewing EEG in longitudinal or transverse bipolar montages became clinical standard. To better observe signals from the temporal lobe, e.g. IEDs, the IFCN recommended to include more inferior electrodes and record from a minimum of 25 electrodes in 2017 (13). In addition to bipolar montages that depict the scalp potential gradients over the upper head, the common average reference montage (CA) of these 25 electrodes was proposed for additional EEG overview (30). All these montages, however, look only at the voltage distribution over the surface of the head.

Hjorth's source derivation (31) was the first attempt to take the EEG from scalp into depth by using a linear transformation corresponding to a simplified Laplacian operator that subtracts the signals of all surrounding electrodes from that at the center electrode. Nowadays, using spherical-splines interpolation (32), the Laplacian or current-source-density (CSD) distribution over the scalp can be estimated more accurately including the boundaries of the electrode array (26). CSD measures the currents flowing from the brain into the scalp through the skull. Thus, CSD maps represent a smoothed reconstruction of the voltage distribution on the brain surface as proposed previously by Freeman (33). Although CSD deblurs the EEG to some extent (34, 35), it faces the same problem as cortical grid recordings of correctly localizing the origin of the underlying oblique dipolar maps. Furthermore, CSD maps are noisier than average-referenced EEG and more difficult to read.

Attempts to take the EEG review beyond the scalp and cortical surface into the brain have been rare but successful by using so-called source montages (20, 26, 30, 36–39). As documented in these papers and illustrated in Figures 6, 9–12, 15, standard source spaces based on MDS models can (a) render the signals of focal brain activities more precisely, i.e. with less contamination from other brain areas, (b) indicate where their origin is located, and (c) demonstrate that the separation is adequate by showing that no or only little cross-talk from other brain regions is seen in the source waveforms (2, 37).

The separation of different brain activities can be optimally tuned by using individual dipole source configurations with equivalent dipoles fixed to known brain structures or fitted to data with high signal-to-noise ratio (SNR). However, dipole orientations in individual MDS models are the key to separate earlier from later source components. The orientation of each dipole has to be fitted to match the recorded scalp maps at time points when the other sources are relatively inactive. Tuning is done using the source waveforms as control to check that cross-talk from earlier onto later sources is minimal, as seen for example in Figures 5–9, 14, 15. Finding the orientation of an IED onset dipole is relatively easy, but finding the orientation of subsequent dipoles to model the regions involved in propagation becomes more complicated, the more dipoles are comprised in the model (11, 12, 25).

The distance between the different active regions in the presented case of cortical reflex myoclonus was just sufficient to prevent linear dependence between the dipole topographies of the forward model (Figure 15, right), because 4 dipoles could be used to separate the postcentral somatosensory (N20, P22) and precentral motor components (MC 4, MC 6) instead of just one regional source with 3 dipoles that would have been quite an accurate model for this region. This separation was only possible by sequentially adapting orientations to minimize cross-talk between source waveforms as seen by the flat onset phases prior to the rising of each activity and the lack of large activities following N20 and P22 (Figure 15, black arrows). If the linear inverse operators were inadequate, activities would spread onto the other sources, as seen in standard source space when generators are between the model sources (Figure 6) or not individually oriented (Figures 9, 15). In contrast, the separation of the source activities from the more distant central SMA and left motor cortex was not critical. Thus, the linear inverse using 6 dipoles could clearly depict the biphasic discharge patterns of the sources of SEP and SEF related to the propagation from primary sensory to primary motor areas, and further on to SMA and contralateral MC (Figure 15).

For the spontaneous jerks in this case of cortical reflex myoclonus, propagation of a typical spike pattern from SC to MC could be documented with inward orientation of the source dipoles into the cortex as expected for IEDs. In addition, the afferent nerve conduction time of 18 ms as defined by the latency of N20 and the efferent conduction times as defined from the source peaks in MC relative to the EMG peaks (MC R to FDIL: 17–18 ms; MC L to FDIR: 19 ms) were highly similar. In contrast, the first activity seen in the averaged spontaneous jerks occurred 6 ms earlier in SC, 22 ms prior to the jerk, i.e. at the same relative latency at which the postcentral N20 occurred prior to the jerks elicited by stimulation. Thus, the spontaneous myoclonic IEDs appeared triggered by the activity in SC and not by MC. A similar time lag of 6 ms between SC and MC has been reported previously for tibial nerve SEPs recorded epi-cortically (40). In fact, when looking at the wideband EEG source waveforms, a rhythmic buildup over 1–2 cycles prior to the IED could be seen in SC, but not in MC (Figure 14, right top).

This imaging of propagation was possible, because the models of the 4–6 discrete dipoles were overdetermined and stable, not only in EEG, but also in MEG. The better spatial resolution of MEG due to the larger number of recording channels having precise relative locations, seemed to compensate for the lack of the “radial” current dimension in MEG, since the SEF model could separate 6 source waveforms highly similar to the SEP model. Thus, we could decompose the complex signals in the surface SEP and SEF into six similar, yet successive biphasic patterns with an overlap becoming progressively more severe with increasing latency. Separation was consistent between EEG and MEG and comparable to the separation of the source waveforms underlying the jerk-locked averaged activity. Due to the lack of inferior electrodes, the activities of the afferent and efferent nerve volleys were not resolved, although the standard SEP montage in Figure 15 suggested such activity in the brainstem trace (BS) prior to the left-hand jerks.

The presented cases illustrate the power of discrete sources in taking the EEG back into the brain when using individually adapted equivalent dipoles to separate the different brain activities underlying evoked potentials and focal IEDs in line with several previous studies (2, 25, 41–43). During EEG and MEG review, however, sources are not known a priori. Hence, standard source spaces covering the whole brain are required to separate the activities of the different regions (16, 26, 37). As shown in Figures 10, 11 and previously (20, 26, 37–39), source montages provide a more focal view, approximate localization and reduction of overlap from other sources. Thus, abnormal signals like IEDs can be recognized more easily. This was confirmed quantitatively by the focality measure introduced here.

After a few similar IEDs have been found, they can be averaged and their spatial vectors, derived from source localization of a regional probe source or from PCA (Figure 6), can be combined with the standard sources. This creates a linear inverse proving focality, if only little activity is contributed by other regions (cf. hypotheses in Figure 2). This principle of reverse source imaging (RSI), applied e.g. by minimizing the cross-talk while moving the probe source, has not yet been exploited and promises to become a very helpful adjunct to source localization of IEDs in the future.

Source montages are based on fixed MDS models (26, 37). Thus, they define a time-invariant linear inverse transformation to take the EEG back into the brain on an approximate, macroscopic level. Because sources are fixed, it is the resulting compound activities of the source waveforms (12, 26, 37) that fully contain the dynamic evolution of the source activity in each brain region. There is no restriction on their temporal dynamics whatsoever; source waveforms are simply linear combinations of the recorded EEG or MEG signals like traditional montages. Being unconstrained, the source waveforms are the result of our testing whether the hypothesized source configuration can explain the data adequately. Thus, the source waveforms reveal immediately when in time an MDS is decomposing the data appropriately and when cross-talk or interference might be occurring.

For example, the decomposition of the SEP in Figure 15 was critical when trying to separate the activities of the posterior (area 3b: N20) and anterior walls (area 4: P25) of the central fissure, because the model had to include two closely located dipoles with almost anti-parallel orientations. Despite this near-to-linear dependence, the onset-to-peak phases of N20 and P25 could be well separated as documented by little crosstalk between all sources prior to the peak of P25 and by the fact that the smaller N20 dipole could be localized accurately to the postcentral gyrus in the onset phase. It remained fixed and displayed the same N20 peak when more sources were added to the EEG model. The second upward peak in the N20 source waveform, however, increased in comparison to the MEG source waveform of M20 since it interacted with the downward peak following P25—an indication that the activation of sensorimotor cortex had become more complex at this later time when more sources were involved. Here, the macroscopic linear decomposition of this SEP data recorded with 29 electrodes had come to its limits.

In contrast, spatio-temporal dipole modeling (STDM) and dynamic causal modeling (DCM) parametrize the waveforms of the compound source activities in addition to source locations and orientations in order to reduce the overall number of unknown parameters and to impose temporal constraints. Whereas, the first STDM studies used empirical biphasic or bi-peaked waveshapes (1, 7), the compound source activities of the more recent DCM methods (44) are based on a physiologically informed network model of the underlying sources. In any case, the goal is to find a model with relatively few temporal and spatial parameters. As shown previously (1, 7), STDM parameters can be estimated in a robust way even when using only few recording channels, because the abundance of the spatio-temporal information is reduced to a small number of parameters far below the degrees of freedom in the data (10). Estimation of these parameters, however, is complicated, because spatial and temporal parameters are severely dependent in a non-linear way.

STDM is based on the assumption that “the neural substrate generating the surface evoked potentials can be defined as consisting of a limited number of neural subsets (generators)…” (1). The activity of each generator—“stationary, as is the spatial organization of the underlying neural structure”—is described by “an equivalent dipole located in, or in close proximity to the neural substrate” and the “temporal course of dipole magnitude is thought to depict the compound discharge processes of the underlying structure.” The generators add “linearly to the scalp potential according to the laws of electrostatics (spatio-temporal superposition)” and their contribution on the scalp can be approximated by choosing “a particular head model” (1). These spatial assumptions also apply to DCM and MDS. STDM and DCM, however, do not estimate the source activities by a simple linear transformation. In addition to the 5 spatial parameters required for each dipole source having unit magnitude (i.e. 3 locations and 2 orientations), the time-varying dipole magnitude is modeled in STDM by e.g. 4~10 parameters describing peak amplitudes, onset, peak and offset times. When applying STDM to AEPs, the strong data reduction allowed for the separation of 6 components of the afferent activity in the brainstem—redefining the origin of the brainstem AEP (7)—and of two components bilaterally in the auditory cortex (1), despite the availability of only 12 scalp EEG channels.

The assumptions underlying STDM are also valid for IEDs. IEDs are generated by a small number of connected regions, i.e. a network with a limited number of nodes. Each underlying cortical patch can be modeled by a fixed equivalent source with a relatively simple bi- or triphasic waveshape as seen in the source waveforms of Figures 12–14. Thus, the propagation around a gyrus and to neighboring areas as shown by the MEG decomposition of a frontal spike in Figure 13, could be resolved with more stability, if the typical spike waveshapes were modeled by a few temporal parameters in addition to the 5 spatial parameters of each equivalent dipole. This would lead to an enormous reduction in degrees of freedom and, thus, STDM appears quite promising in providing a robust separation of the components underlying averaged IEDs. However, software to adapt such an STDM model to IEDs is currently not available. Neither has DCM been applied to focal, lesion-related IEDs, to our knowledge, possibly due to the computationally very demanding Bayesian methods required by DCM (44). However, DCM models have been applied to understand the networks underlying electrographic seizures using EEG/ECoG (45).

MDS models are also the key to measure connectivity between brain regions (46). The strong overlap due to volume conduction makes most scalp signals highly correlated, as evidenced by the focality measure introduced here. As illustrated above, a linear inverse can be constructed to isolate the activities of two brain regions without any contamination by mutual volume conduction, if the regularization coefficients of the two regions are set to zero. Thus, their mutual cross-talk is zero. Their connectivity can be assessed, if the other brain regions are modeled by a standard source space. Cross-talk of the other regions is projected onto the two sources of interest with the same phase. This interference can be removed using out of phase coherence (47). For connectivity analysis, one ideally constructs specific source spaces using prior information from multi-modal functional imaging and individual structural MRI (48).

To conclude, taking the EEG back into the brain using a standard or specific source space is a prerequisite to analyze the networks underlying IEDs and evoked- or event-related potentials. If the results of reviewing the EEG in standard source space are inconclusive, functional and structural information from other modalities should be used to create more specific individual source spaces.

## Materials and Methods

### Subjects and Data

The presented EEG and MEG examples were available as digitized, anonymized data from past studies in various evoked potential laboratories and epilepsy units at the Max-Planck-Institute for Psychiatry in Munich (1, 7, 10, 17), at the Kohnan Hospital and Tohoku University of Sendai (49), and at the University Hospitals of Aarhus (16), Heidelberg (25, 26, 50), Iowa (51), and Munich (6). For all studies, informed consent of the subjects and approval of the local ethics committees had been obtained.

### Software and Digital Signal Processing

Digital signal processing was performed using BESA Research 7.0 (BR7), BESA MRI 2.0 (BM), and BESA Simulator 1.4 (BESA GmbH, Gräfelfing, Germany). Standard MDS models were created by seeding sources into the standard MRI of BR7 in Talairach space within the source analysis module of BR7. Individual MDS models were created using sequential fitting strategies (11, 25) while visually minimizing cross-talk between source waveforms. Individual source locations as well as LORETA and CLARA images were visualized in Talairach space using the source analysis module of BR7 and structural T1-weighted MRI images were rendered using BM.

Using specific batch functions, individual and standard source montages were saved to be applied effectively while inspecting continuous EEG data using a digital zero-phase-shift filter of 2–35 Hz to reduce artifacts and enhance perception of IEDs (26). IEDs were averaged using wideband filter settings in the ERP module of BR7 and the averages were filtered with a forward low filter of 5 Hz to obtain a clear baseline prior to onset and a zero-phase-shift high filter at 40 Hz to reduce artifacts of higher frequencies. Averaged evoked potential filter settings depended on the time range of the observed components and are specified in the figures.

### Source Space Transformations

#### Scalp and Source Montages Are Linear Transformations

EEG data can be described as a matrix D_r_ with as many rows as recorded channels. Each column contains the recorded voltages at one sampling point in time. EEG data is typically recorded against a common reference that is often defined by a hardware average of the signals from two or more recorded electrodes, e.g. F3 and F4. Thus, signals are biased, because the voltage difference is small to nearby and large to remote electrodes. To remove this bias, bipolar montages are created by subtracting neighboring channels. In the average reference montage, bias is removed by subtracting the signal averaged over all channels from each channel. Thus, bipolar montages measure the voltage gradients along the scalp in longitudinal or transverse directions while the average reference comes close to showing the “true” voltage at each electrode, if the electrodes cover the upper and lower head with sufficient equidistant spacing (26).

These montages are special forms of linear combinations, defined by multiplying the recorded matrix D_r_ with a linear operator B to create a bipolar montage D_B_ or with A to create the average reference matrix D:

(1) $$\begin{array}{l} {\text{D}}_{\text{B}}=\text{B }{\text{D}}_{\text{r}} \end{array}$$

(2) $$\begin{array}{l} \text{D}=\text{A }{\text{D}}_{\text{r}} \end{array}$$

Similarly, the signals in source space are described by a matrix S using a special inverse linear transformation matrix T^−1^:

(3) $$\begin{array}{l} \text{S}={\text{T}}^{-1}\text{ D}={\text{T}}^{-1}\text{A }{\text{D}}_{\text{r}} \end{array}$$

Each row of A, B or T^−1^ contains the weight factors used to multiply the recorded channels in order to obtain one channel in the bipolar, average referenced or source montage. The weight factors of A and B have been published (26). For example, a bipolar channel simply uses a weight factor of +1 for the positive, −1 for the negative, and 0 for the other channels. The inverse matrix T^−1^ has more specific, non-zero factors for each recorded channel. For example, T^−1^ of Figure 7 has 4 rows, one for each source, and 12 columns with the source-specific factors for each recorded channel.

How can we calculate T^−1^, i.e. the different weight factors needed to reconstruct each channel in the individual and standard source spaces from the recorded channels?

#### Linear Overlap: The Forward Model With Fixed Sources

According to the laws of physics, the signals of all sources in the brain (rows in S) overlap linearly at the scalp to form the EEG together with some remaining noise, i.e. the signals not explained by the chosen set of discrete sources (noise matrix N):

(4) $$\begin{array}{l} \text{D}=\text{T S}+\text{N}=\text{A L S}+\text{N} \end{array}$$

The contribution of each source to the EEG is defined by its voltage map on the scalp. Thus, the columns of the topography matrix T contain the average-referenced maps due to unit currents at each source. Using a volume conductor model of the head (forward model), one can predict the reference-free scalp maps of each source by calculating matrix L, the so-called leadfield vectors (26). To equate the average-reference matrix D with the predicted voltages, we must apply A also onto L to obtain T. The amount of signal contributed to the EEG signals d_i_ (t) (rows *i* in D, *i* = 1….nchans) at each point in time by source k is given by the magnitude of the (still unknown) source waveform signals s_k_ (t) (rows k in S, *k* = 1….ns) multiplied with the fixed, time-independent topography vector k, i.e. the column k of T.

#### The Inverse Linear Operator: Individual and Standard Source Spaces

After having defined matrix T by a specific forward model, one can calculate S by applying the linear inverse T^−1^ onto Equation (4) from the left:

(5) $$\begin{array}{l} \text{S}={\text{T}}^{-1}\;{\text{D}-\text{T}}^{-1}\text{ N} \end{array}$$

since the product of the forward and inverse matrices is the identity matrix I:

(6) $$\begin{array}{l} \text{T }{\text{T}}^{-1}=\text{I} \end{array}$$

Since T is not a square matrix, T^−1^ is given by the Moore-Penrose pseudoinverse (41). Prior to inversion, the forward column vectors of T are normalized to avoid bias against deep sources in the brain and to enable noise reduction and smoothing by regularization (Figure 9):

(7) $$\begin{array}{l} {\text{T}}_{\text{N}}=\text{T }{\text{W}}^{-1} \end{array}$$

The diagonal in W contains the root-mean-square magnitudes of each topography vector and the norm of each column in T_N_ is 1. The pseudoinverse of T_N_ is given by

(8) $$\begin{array}{l} {\text{T}}_{\text{N}}^{-1}={\left({\text{T}}_{\text{N}}^{\text{T}}{\text{T}}_{\text{N}}+\text{R}\right)}^{-1}\;{\text{T}}_{\text{N}}^{\text{T}} \end{array}$$

The correlation matrix of the topographies (${\text{T}}_{\text{N}}^{\text{T}}$ is the transpose of T_N_) is the kernel of the inverse with values of 1 in the main diagonal. In individual source spaces with few sources (ns < nchans) the regularization matrix R can be set to zero. Thus, source activities are not smoothed and there is no cross-talk between sources, because applying ${\text{T}}_{\text{N}}^{-1}$ from the left onto T_N_ produces the identity matrix, i.e. each signal is rendered maximally while spread from the other sources is suppressed (20).

In standard source spaces with more source dipoles (ns ~ nchans), the coefficients in the diagonal regularization matrix R can be set specifically for each source to a small percentage of 1 as, for example, to 1.2% in source space 25. This results in little cross-talk and moderate smoothing as can be seen in Figures 6, 8–12. Furthermore, the recorded data matrix D_r_ is first projected onto 81 standard electrodes by the linear operator P using spherical splines interpolation as for mapping (26). This projection is also very convenient, if a noisy recording channel has to be excluded, because the leadfields L can be pre-calculated for the 81 standard-electrodes using the standard sources.

In summary, the EEG is taken back into the brain by a single linear inverse operator, if we combine the series of linear operations into a single linear transformation matrix M acting on the recorded data:

(9) $$\begin{array}{l} S=M\;D_{\text{r}}=\left(W^{-1}T_{\text{N}}^{-1}P\;A\right)D_{\text{r}} \end{array}$$

P is the unity matrix I in discrete individual source models and the spline-interpolation matrix (dimension: 81^*^nchans) in standard MDS models. In distributed source models (ns >> nchans), the inverse is calculated in sensor and not in source space (14), because the number of sources is too large for inversion in source space. This leads to more smoothing. Furthermore, regularization with different coefficients for specific sources is impossible. When inverting in source space, however, ECG components are removed completely, if their regularization coefficients are set to zero (15), and source activities of a particular brain region can be rendered without cross-talk to other regions.

As shown above, equivalent dipoles and regional sources have a high accuracy in modeling the activity of a relatively large brain area. In addition, the linear inverse T^−1^ is minimizing the noise term N in Equation (5) as it represents an implicit least-squares-fit of the source waveforms S to the data D. Therefore, a small number of dipoles in the individual source spaces and 25 regional sources in standard source space 25 were sufficient to model D with little noise in all cases presented here. Typically, residual variance (RV) in standard source spaces was below 1%, i.e. the noise projected by matrix N onto the different source regions was small.

### Focality in Source and Scalp Space

Focality was defined to measure the spread of activity over the channels in scalp vs. source space as follows: In each space, the channel having maximum signal was determined within a selected epoch (IEDs: −50: +150 ms relative to peak). Next, the maximum signal was correlated with itself and all other channels to obtain a vector of squared covariance coefficients sorted by magnitude and normalized to 100%. Cumulative focality was calculated by summing the magnitudes from the largest to the n-largest values to obtain F(n) as illustrated in Supplementary Figure 1.

## Data Availability

The raw data supporting the conclusions of this manuscript will be made available by the authors, without undue reservation, to any qualified researcher.

## Ethics Statement

The presented EEG and MEG examples were available as digitized, anonymized data from past studies in various evoked potential laboratories and epilepsy units at the Max-Planck-Institute for Psychiatry in Munich (1, 7, 10, 17), at the Kohnan Hospital and Tohoku University of Sendai (48), and at the University Hospitals of Aarhus (16), Heidelberg (25, 27, 49), Iowa (50), and Munich (6). For all studies, informed consent of the subjects and approval of the local ethics committees had been obtained.

## Author Contributions

MS analyzed the EEG and MEG data and designed all figures. PB performed simulations and specific software developments for this study and critically reviewed the manuscript. NN and SB provided the data from their epilepsy units, contributed to data interpretation, and critical revision of manuscript and figures.

### Conflict of Interest Statement

MS is shareholder and employee of BESA GmbH, a company developing software for advanced EEG and MEG analysis. PB is a free-lance employee of BESA GmbH. The remaining authors declare that the research was conducted in the absence of any commercial or financial relationships that could be construed as a potential conflict of interest.

## Supplementary Material

Supplementary Figure 1 Assessment of focality: Nine IEDs (1-s segments) are shown in scalp space (middle) and standard source space 25 (top). The visual impression of higher focality in source as compared to scalp space, i.e. the IED signal spreads less and with smaller magnitude over the channels in source than scalp space, is quantified by the new measure of focality F(n) summing the cumulative variance of the correlation with the largest signal from the largest to the n-th largest channel (see Materials and Methods). The focality diagrams at the bottom show focality in % for segments 2 (left) and 5 (middle). In segment 2, focality is considerably larger in source space at lower n-values while the more widespread IED in segment 5 exhibits similar focality in source and scalp space. The diagram at the lower right depicts source focality together with the difference of focality in source vs. scalp space over the 65 left temporal IEDs of the mTL patient shown in Figure 11. The difference is >0 in all but 2 IEDs. This proves the much higher focality in source space (*p* < 0.001).
