# Supplementary figures and images for: Taking the EEG Back Into the Brain: The Power of Multiple Discrete Sources
Source: Front Neurol. 2019 Aug 20;10:855. doi: 10.3389/fneur.2019.00855 (PMC6710389; doi:10.3389/fneur.2019.00855)

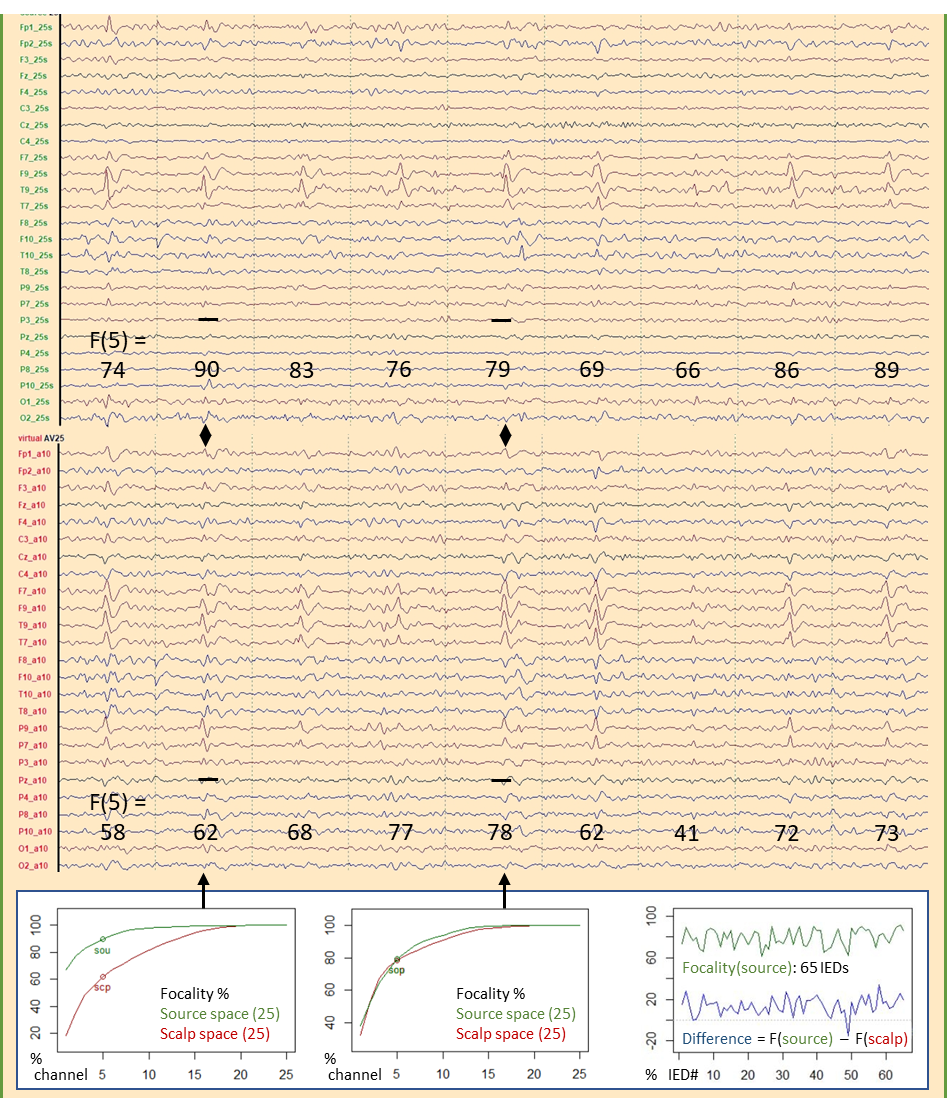

Supplement: Supplementary Figure 1 — Assessment of focality: Nine IEDs (1-s segments) are shown in scalp space (middle) and standard source space 25 (top). The visual impression of higher focality in source as compared to scalp space, i.e. the IED signal spreads less and with smaller magnitude over the channels in source than scalp space, is quantified by the new measure of focality F(n) summing the cumulative variance of the correlation with the largest signal from the largest to the n-th largest channel (see Materials and Methods). The focality diagrams at the bottom show focality in % for segments 2 (left) and 5 (middle). In segment 2, focality is considerably larger in source space at lower n-values while the more widespread IED in segment 5 exhibits similar focality in source and scalp space. The diagram at the lower right depicts source focality together with the difference of focality in source vs. scalp space over the 65 left temporal IEDs of the mTL patient shown in Figure 11. The difference is >0 in all but 2 IEDs. This proves the much higher focality in source space (p < 0.001). [file Image_1.TIFF]
